# Auxin dysregulation: a key early event in sugarcane susceptibility to *Sporisorium scitamineum*

**DOI:** 10.1186/s12870-026-08187-5

**Published:** 2026-01-24

**Authors:** Xin Hu, Zhengying Luo, Shenglin Ren, Jing Zhang, Chaohua Xu, Chunjia Li, Xinlong Liu

**Affiliations:** 1 State Key Laboratory of Tropical Crop Breeding, Yunnan Academy of Agricultural Sciences, Kunming, 650205 P.R. China; 2 Yunnan Key Laboratory of Sugarcane Genetic Improvement, Key Laboratory of Sugarcane Biology and Genetic Breeding (Yunnan), Ministry of Agriculture and Rural Affairs, Sugarcane Research Institute, Yunnan Academy of Agricultural Sciences, Kaiyuan, Yunnan 661699 China; 3 The Key Laboratory for Crop Production and Smart Agriculture of Yunnan Province, College of Agronomy and Biotechnology, Yunnan Agricultural University, Kunming, 650201 China

**Keywords:** Sugarcane, Smut, IAA, Virulence, Effector, Indole-3-acetaldehyde dehydrogenases

## Abstract

Sugarcane smut, caused by *Sporisorium scitamineum*, severely limits global sugarcane yield. Although auxin manipulation is a recognized virulence mechanism in plant-pathogen interactions, biological basis and mechanisms in orchestrating host colonization within the sugarcane-smut pathosystem remain elusive. This knowledge gap constrains a mechanistic understanding of auxin-mediated pathogenesis, thereby limiting opportunities to design auxin-based control strategies. Through integrated approaches combining exogenous hormone treatment, physiological profiling, and spatiotemporal gene expression analysis in susceptible and resistant genotypes, we aim to reveal how IAA coordinates an early host-pathogen dialogue. Our findings showed that IAA promoted infection in a dose-dependent manner: it reprogrammed host physiology at low concentrations in planta while inhibiting fungal growth at higher levels in vitro. IAA-mediated susceptibility involved disrupted redox homeostasis through suppressed activities of *SOD* and *CAT*, altered flavonoid metabolism, and upregulation of fungal effector genes such as *Tin2* and *Nkd1*. Expression analysis further revealed temporally and genotype-dependent regulation of IAA-biosynthetic genes, with strong late-stage induction of *SsIad13* and *SsIad16* exclusively in susceptible plants. The resistant genotype demonstrated a superior ability to maintain redox balance and sustain flavonoid accumulation, underscoring the importance of constitutive host defense in limiting disease progression. Together, this study establishes auxin signaling as a key virulence module in *S. scitamineum*, synchronizing fungal IAA production, host immunity suppression, and effector deployment. These insights advance our understanding of smut disease mechanisms and propose fungal IAA pathway elements as candidate targets for future resistance breeding.

**Supplementary Information:**

The online version contains supplementary material available at 10.1186/s12870-026-08187-5.

## Introduction

Sugarcane (*Saccharum* spp. hybrids) is the principal commercial sugar crop, dominating global production with a contribution of approximately 80%. Sugarcane smut, caused by the fungal *Sporisorium scitamineum*, is the most devastating disease that substantially compromises yield in sugarcane-growing regions worldwide. The disease is characterized by the emergence of black whip-like structures from the shoot apex, often occurred by morphological alterations such as internode elongation, chlorotic and slender leaves, and excessive tillering [1, 2]. Disease severity intensifies with successive ratoon cycles, and epidemics can lead to severe losses in both cane yield and sucrose content [3, 4]. The pathogen typically invades through bud tissues, and susceptibility increases upon removal of bud scales or via needle inoculation, underscoring the role of structural barriers in early defense [5, 6]. Although fungal penetration occurs within days, a prolonged latent period of 2–3 months precedes symptom appearance, rendering the early infection phase critical for host resistance [7, 8]. In response to invasion, sugarcane activates a multi-layered defense system involving cell wall fortification (e.g., lignin deposition), synthesis of antimicrobial secondary metabolites (e.g., phytoalexins and flavonoids), reactive oxygen species (ROS) burst, MAPK pathway activation, and hormonal reprogramming [9–11]. Phytohormones are central to the regulation of these immune responses. In addition to the classical defense hormones—salicylic acid (SA), jasmonic acid (JA), and ethylene (ET)—growth-related hormones such as auxins, cytokinins, brassinosteroids, abscisic acid, and gibberellins constitute essential elements of the immune signaling network [12, 13]. During sugarcane–smut interaction, the activity of defense enzymes serves as a key indicator of resistance. Enzymes involved in secondary metabolism, including phenylalanine ammonia-lyase (PAL) and tyrosine ammonia-lyase (TAL), exhibit elevated activity in resistant genotypes and contribute to the biosynthesis of defensive compounds [14, 15]. Pathogen infection also induces ROS accumulation and membrane lipid peroxidation, activating antioxidant enzymes such as superoxide dismutase (SOD), peroxidase (POD), and catalase (CAT), while increasing malondialdehyde (MDA) levels. Resistant cultivars rapidly accumulate H₂O₂ at infection sites, triggering localized cell death and limiting pathogen spread [16, 17].

Auxin, primarily represented by indole-3-acetic acid (IAA), regulates essential developmental processes including cell elongation and apical dominance. Beyond its growth-related functions, IAA plays complex, context-dependent roles in plant–pathogen interactions, serving both as a host signaling molecule and a microbial virulence factor [18, 19]. The effect of auxin on disease outcome is influenced by pathogen lifestyle: it bolsters resistance against necrotrophs such as *Alternaria* and *Rhizoctonia*, and certain viruses, by enhancing JA signaling and ROS production, while increasing susceptibility to biotrophic and hemibiotrophic pathogens like *Pseudomonas syringae*, *Magnaporthe grisea*, and *Agrobacterium tumefaciens* [20–23]. For example, exogenous auxin reduces susceptibility to necrotrophs, whereas auxin signaling mutants exhibit impaired resistance [20, 24]. Conversely, elevated auxin levels or signaling intensify susceptibility to *P. syringae*, and auxin-deficient mutants show enhanced resistance to biotrophs [25, 26].

Pathogens have evolved multiple strategies to subvert host auxin signaling. Biotrophic and hemibiotrophic species often enhance auxin signaling through de novo IAA synthesis or by secreting effectors that target auxin signaling components. For instance, the *P. syringae* effector AvrRpt2 promotes the degradation of AUX/IAA repressors to potentiate auxin responses [27], while *Agrobacterium tumefaciens* induces host auxin biosynthesis to promote gall formation [28]. Plant viruses such as rice dwarf virus (RDV) and tobacco mosaic virus (TMV) disrupt auxin signaling by interfering with AUX/IAA proteins, leading to developmental aberrations and symptom exacerbation [29, 30]. In *P. syringae* PtoDC3000, IAA fine-tunes virulence by repressing *type III secretion system* (*T3SS*) genes and activating transcription factors such as *TvrR*, thereby coordinating infection stages [25]. In *A. tumefaciens*, elevated IAA levels suppress virulence gene expression after gall establishment [31]. A principal mechanism by which auxin promotes biotrophic susceptibility is through antagonism of SA-dependent defense [26, 32], although SA-independent pathways—such as modulation of host nutrient transport—also contribute [25, 33]. Together, these findings underscore the duality of auxin function in plant–pathogen interactions, shaped by both host hormonal cross-talk and direct microbial perception of IAA.

Although the role of auxin has been well characterized in the maize–*Ustilago maydis* pathosystem [34], its involvement in the *S. scitamineum*–sugarcane interaction remains poorly understood. Unlike *U. maydis*, which induces tumorigenic growth in maize, *S. scitamineum* infection in sugarcane leads to the development of long whip-like structures, suggesting species-specific differences in auxin-mediated symptom development [35]. Nevertheless, conserved mechanisms across smut fungi imply a critical role for auxin in this pathosystem. Genomic analyses confirm that *S. scitamineum* possesses a functional IAA biosynthesis pathway, including the tryptophan (Trp) aminotransferase SsAro8—an ortholog of *U. maydis* Tam1—that catalyzes the first step of Trp-dependent IAA production and is essential for mating, filamentation, and biofilm formation [36]. This suggests that fungal-derived IAA may facilitate host colonization by modulating developmental processes. In addition, *S. scitamineum* is predicted to secrete effectors that interfere with host auxin signaling. Although orthologs of known TOPLESS (TPL)-interacting protein effectors (Tips) from *U. maydis* remain unconfirmed in *S. scitamineum*, the conserved role of TPL as an auxin signaling corepressor—often targeted by smut fungi to de-repress auxin-responsive genes [37–39]—suggests that *S. scitamineum* may similarly disrupt TPL function to promote host cell division and elongation, thus facilitating infection.

In this study, we dissected the roles of auxin in the *S. scitamineum*–sugarcane interaction using integrated physiological, molecular, and transcriptional approaches. By employing susceptible and resistant sugarcane genotypes under IAA treatment, we demonstrated that IAA promoted fungal virulence not by directly enhancing pathogen growth, but via large-scale manipulation of host immunity and metabolism. We showed that *S. scitamineum* employs a multi-layered approach to manipulate auxin signaling, involving the dynamic control of fungal IAA biosynthesis, disruption of host redox and secondary metabolism, and coordinated effector expression. Host genetic background profoundly influenced these interactions, underscoring its importance in disease outcome. Our results establish auxin manipulation as a key mechanism of *S. scitamineum* pathogenicity and suggest novel, potential targets for smut-resistant crop breeding.

## Results

### Exogenous IAA promotes susceptibility in sugarcane but directly inhibits *S. scitamineum* growth in vitro

**Fig. 1:**
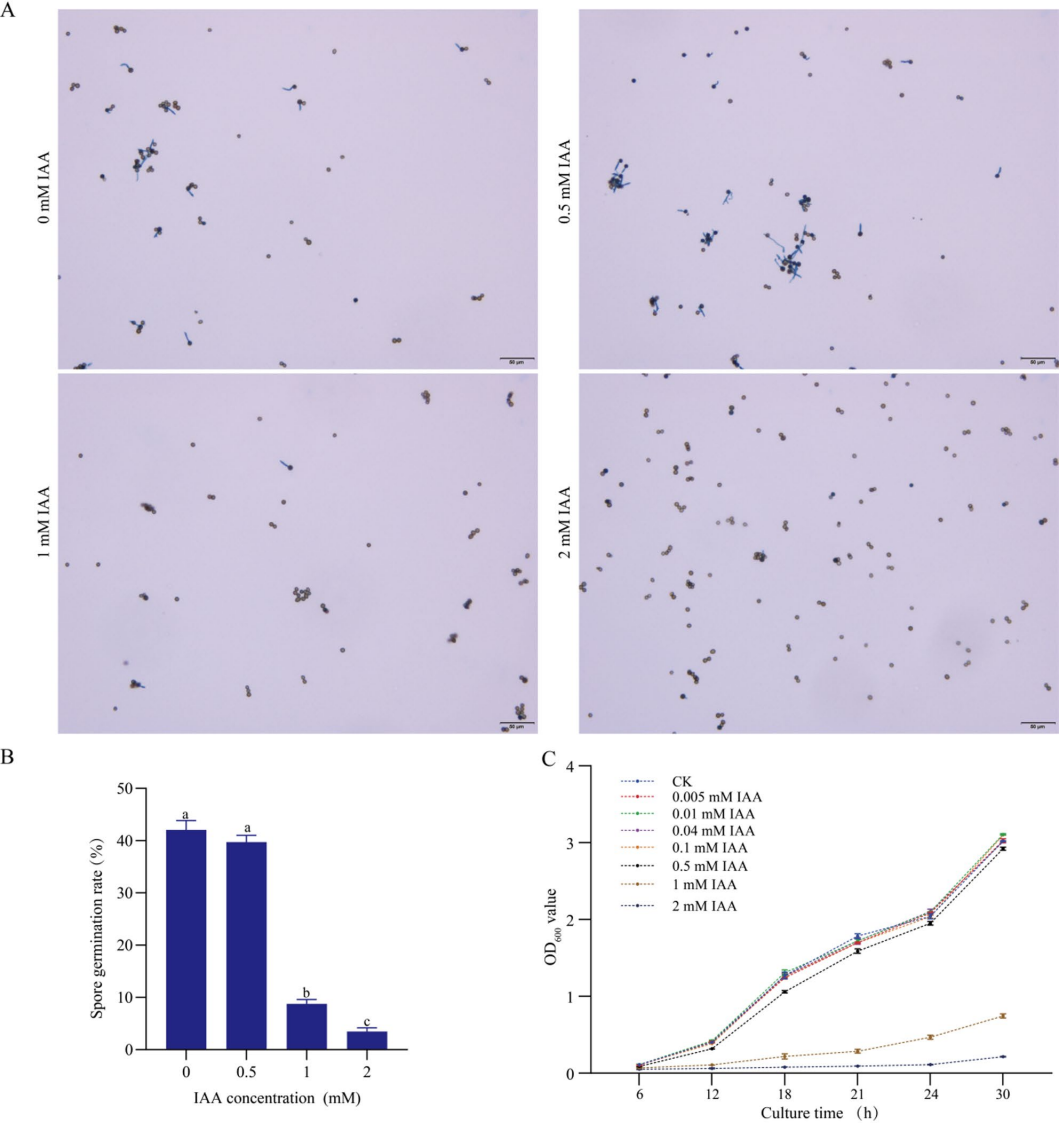
Effects of indole-3-acetic acid (IAA) on spore germination and mycelial growth of *S. scitamineum*. **A** Microscopic observation of spore germination under different IAA concentrations (0, 0.5, 1, 2 mM). **B** Spore germination rates across IAA concentrations. Data represent the mean ± SD from three biological replicates. For each replicate, germination was counted by examining at least 300 spores per biological sample under the light microscope. Lowercase letters indicate statistically significant differences between treatments (one-way analysis of variance (ANOVA) followed by Fisher’s least significant difference (LSD) test; *P* < 0.05). **C** Time-course analysis of mycelial growth measured as OD_600_ under different IAA concentrations. Data represent the mean ± SD of three biological replicates, each biological replicate included twice technical measurements at every time point

To elucidate the role of auxin in the sugarcane-smut interaction, we first examined the direct effect of indole-3-acetic acid (IAA) on the pathogen *S. scitamineum* in vitro. Fungal spores were treated with a gradient of IAA concentrations. As shown in Fig. 1, low IAA concentrations (< 0.5 mM) did not significantly affect spore germination or hyphal growth compared to the untreated control (Fig. 1). In contrast, higher concentrations (1–2 mM) markedly inhibited both germination and filamentous growth in a dose-dependent manner. These results indicate that IAA does not function as a direct growth signal for the fungus at low concentrations but acts as a potent inhibitor at non-physiological levels.

**Fig. 2:**
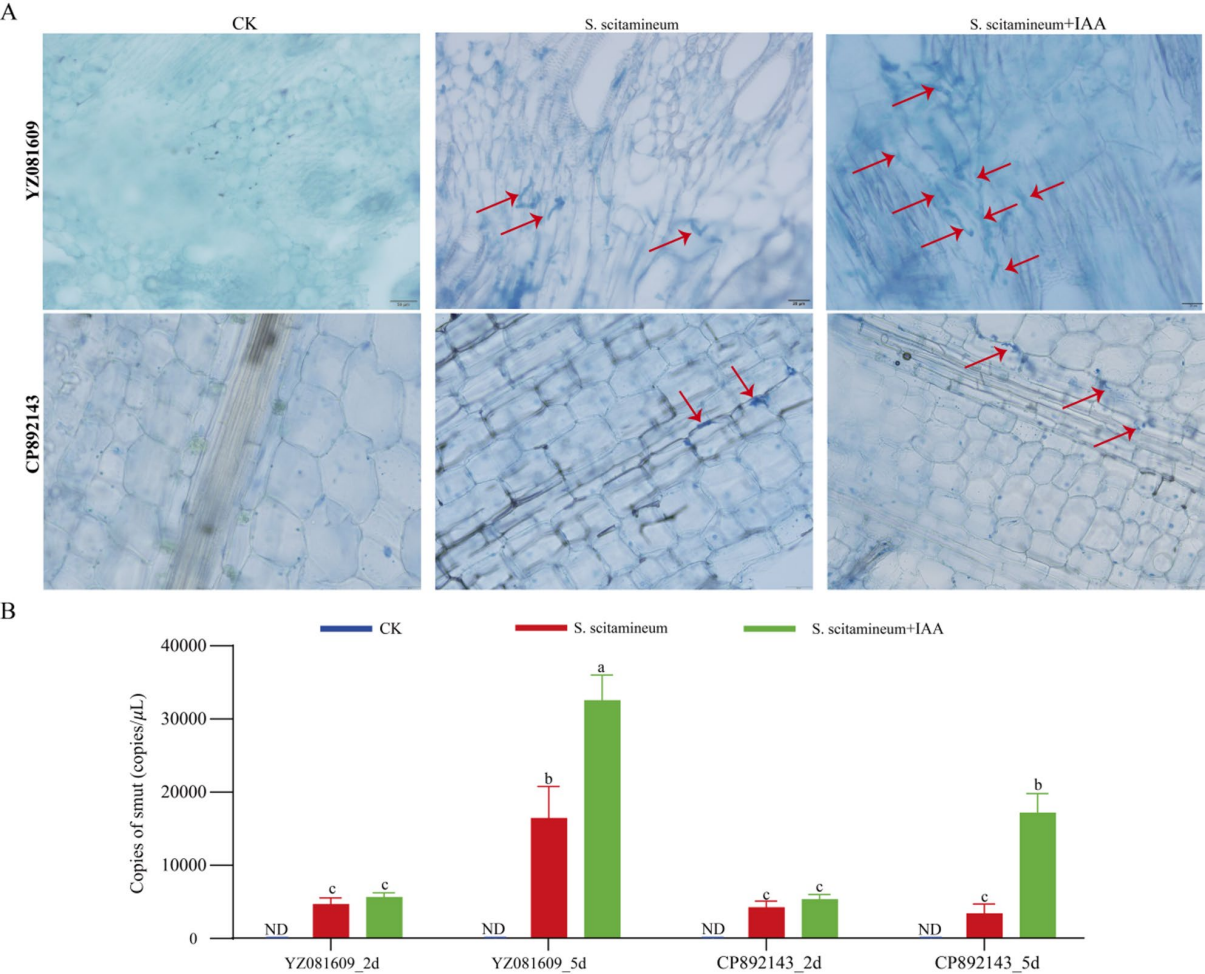
Histological observation and quantification of *S. scitamineum* colonization in susceptible (YZ081609) and resistant (CP892143) cultivars under different treatments. **A** Microscopic images of sugarcane tissue sections stained to visualize *S. scitamineum* (red arrows indicate fungal structures) at 5 days post-treatment. Treatments include control (CK), *S. scitamineum* inoculation, and *S. scitamineum* inoculation + IAA application. **B** Quantification of *S. scitamineum* copies via quantitative real-time PCR (qPCR) in YZ081609 and CP892143 at 2 and 5 dpi. Data represent mean ± SD from three biological replicates with three technical replicates per biological replicate. Different lowercase letters indicate significant differences between groups (one-way ANOVA followed by LSD test; *P* < 0.05). ND, Not detectable

We next evaluated the effect of exogenous IAA on disease development in planta. Susceptible (YZ081609) and resistant (CP892143) cultivars were inoculated with *S. scitamineum* and treated with a low, non-inhibitory concentration of IAA (0.5 mM). Disease progression and fungal biomass accumulation were monitored. In the susceptible cultivar YZ081609, IAA application significantly promoted both the quantity and growth rate of hyphae at 5 days post-inoculation (dpi), resulting in substantially higher infection rates compared to the resistant cultivar (Fig. 2A). In contrast, the resistant cultivar CP892143 maintained strong defense capacity, with no noticeable increase in either fungal colonization density or hyphal extension upon IAA treatment. Quantitative assessment of fungal biomass revealed that IAA-treated plants of both cultivars exhibited higher fungal loads than mock-treated controls at 5 dpi (Fig. 2B). Consistently more extensive fungal colonization was observed in the susceptible YZ081609 than in the resistant CP892143 under both mock and IAA treatments, reinforcing the intrinsic susceptibility of YZ081609 to smut infection. Collectively, these results indicate that IAA exerts a dual role in the sugarcane–smut interaction: it directly inhibits fungal growth at high in vitro, yet enhances disease susceptibility in planta。.

### *S. scitamineum* infection and exogenous IAA perturbed ROS homeostasis

To investigate how *S. scitamineum* infection and exogenous IAA influence host physiological and immune responses, we measured changes in IAA levels and defense-related physiological indicators in susceptible (YZ081609) and resistant (CP892143) sugarcane varieties (Table S1). To systematically evaluate the regulatory effects of variety, treatment, time, and their interactions on sugarcane’s physiological defense mechanisms against *S. scitamineum*, a multifactorial analysis of variance (ANOVA) was conducted (Table 1). The results demonstrated that treatment (Group) exerted highly significant main effects on IAA content, POD, SOD, and CAT activities (*P* < 0.01), underscoring its pivotal role in shaping these physiological responses. Time also exhibited significant main effects on flavonoid accumulation and H₂O₂ levels (*P* < 0.01), indicating distinct temporal dynamics in secondary metabolism and oxidative stress. Moreover, significant Variety × Time interactions were detected for flavonoid content and POD activity (*P* < 0.05), revealing genotype-specific temporal regulation of these defense-related traits.

**Table 1:** Significance overview of multifactorial ANOVA for physiological indicators

Indicator	Main effect(F/*P*)			Interaction Effect (F/*P*)			
	Variety (V)	Group (G)	Time (T)	V × G	V × T	G × T	V × G × T
IAA	0.123/0.729	138.392/0.000***	0.242/0.627	0.015/0.985	1.415/0.246	0.639/0.536	0.045/0.956
Flavonoid	1.694/0.205	2.837/0.078	11.776/0.002**	0.151/0.861	7.686/0.011*	0.620/0.546	1.112/0.345
PAL	1.188/0.287	3.866/0.035*	1.128/0.299	2.790/0.081	0.017/0.896	0.196/0.823	0.136/0.874
POD	2.133/0.157	8.352/0.002**	2.321/0.141	0.074/0.929	4.997/0.035*	1.193/0.321	0.359/0.702
H₂O₂	1.216/0.281	2.559/0.098	9.286/0.006**	0.208/0.814	3.284/0.082	0.957/0.398	0.913/0.415
SOD	0.254/0.619	12.755/0.000***	0.109/0.744	0.750/0.483	1.536/0.227	2.843/0.078	1.945/0.165
CAT	2.375/0.136	9.447/0.001**	0.128/0.724	0.645/0.534	1.774/0.195	0.290/0.751	1.682/0.207

F/P is formatted as F value/P value. Variety: V; Group: G; Time: T. Significance levels: *P < 0.05, **P < 0.01, ***P < 0.001

**Fig. 3:**
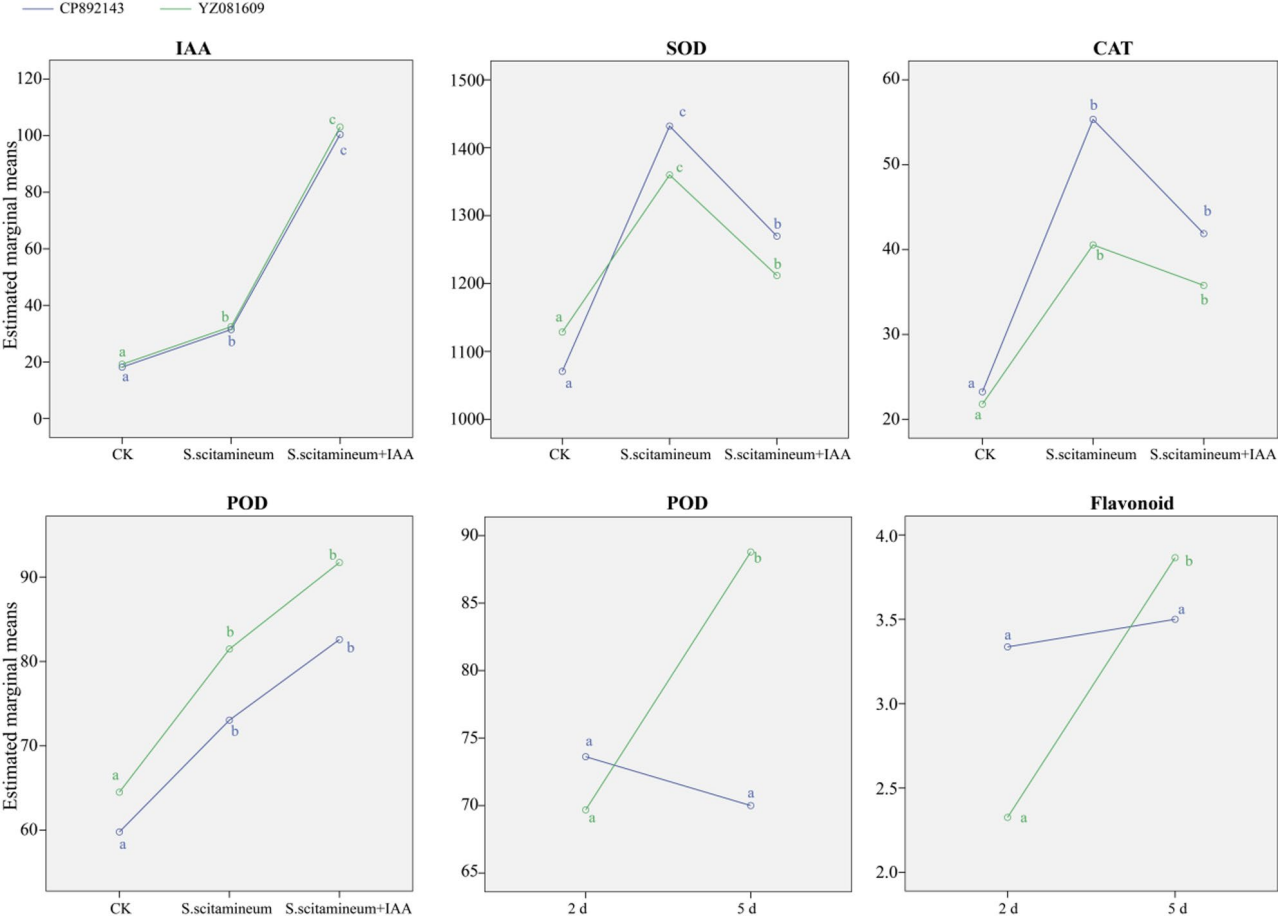
Estimated marginal means of physiological and biochemical indicators in two sugarcane varieties (CP892143, blue; YZ081609, green) under different treatments or time points. Values are expressed per gram fresh weight (FW) or dry weight (DW). IAA (ng·g⁻¹·FW); SOD, CAT, POD, (U·g⁻¹·FW·min⁻¹); Flavonoid (mg·g⁻¹·DW). Data are presented as estimated marginal means from multifactorial ANOVA analysis. Statistical comparisons were performed separately within each color group. Significant differences between groups (*P* < 0.05) were determined by LSD test and are indicated by different lowercase letters (a, b, c)

As shown in Fig. 3, the estimated marginal means of key physiological indicators varied substantially among treatments and varieties. For IAA, SOD, CAT, and POD—parameters strongly influenced by treatment—both *S. scitamineum* inoculation and the combined inoculation with IAA consistently elevated their levels relative to the control across both genotypes. The *S. scitamineum* + IAA treatment further enhanced IAA accumulation but significantly reduced SOD activity compared to pathogen-only infection, suggesting that IAA suppressed certain antioxidant responses. Significant Variety × Time interactions revealed genotype-specific temporal patterns in flavonoid content and POD activity. The resistant cultivar CP892143 sustained elevated flavonoid accumulation at early infection stages, maintaining relatively stable levels throughout the experimental period. In contrast, the susceptible YZ081609 displayed more pronounced induction of flavonoid content and POD activity over time. This differential regulation underscores CP892143’s more constitutive defense strategy compared to the induced but delayed response in the susceptible genotype. These findings collectively demonstrated that *S. scitamineum* infection significantly disrupted host ROS homeostasis and flavonoid biosynthesis, and that exogenous IAA promoted abnormal IAA accumulation while suppressing the activity of antioxidant enzymes like SOD, thereby compromising the plant’s oxidative stress defense capacity.

### IAA linked oxidative stress with secondary metabolism in shaping treatment-specific physiological patterns

**Fig. 4:**
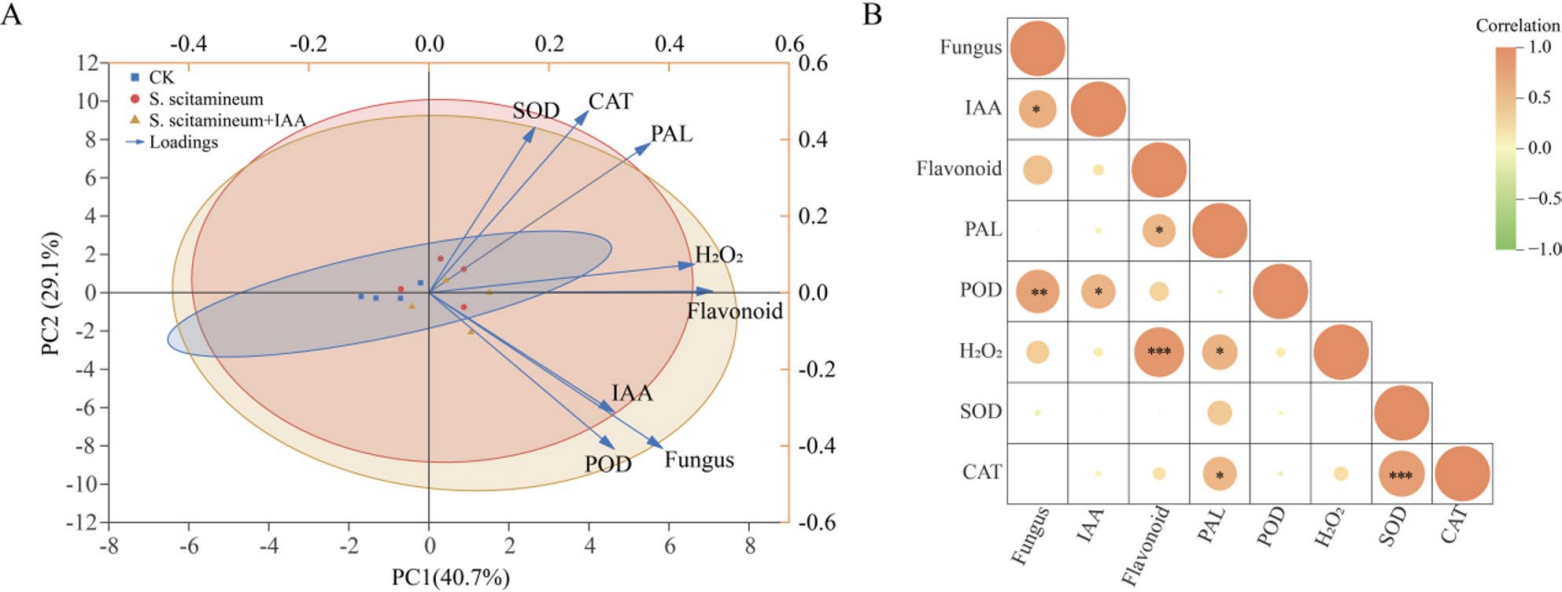
PCA and correlation analysis of physiological and pathological indices in sugarcane under different treatments. **A** PCA plot showing the distribution of control (CK, blue squares), *S. scitamineum*-inoculated (red circles), and *S. scitamineum* + IAA-treated (yellow triangles) groups. Blue arrows represent variable loadings (SOD, CAT, PAL, H₂O₂, flavonoids, IAA, POD, fungal biomass). **B** Correlation heatmap of variables. Color intensity and circle size represent Pearson correlation coefficients (red: positive, green: negative), with significance determined by two-tailed tests (*P < 0.05, **P < 0.01, ***P < 0.001)

Principal component analysis (PCA) revealed that PC1 (40.7%) and PC2 (29.1%) together explained 69.8% of the total variance, effectively capturing core differences among samples and variables (Fig. 4A). The control group (CK) clustered separately in the negative PC1 region, whereas the *S. scitamineum*-inoculated and inoculated + IAA groups shifted toward positive PC1, indicating distinct physiological changes following inoculation. The inoculated group (red circles) showed a dispersed distribution, reflecting individual variation in plant responses to infection. The inoculated + IAA group shifted further toward negative PC2, suggesting that exogenous IAA amplified the shifts induced by inoculation. Key variables driving these patterns included defense-related metabolites (PAL, flavonoids, H₂O₂), which were positively correlated with PC1. The CK group was distanced from these variables, indicating their upregulation in both inoculated and inoculated + IAA groups. In contrast, IAA, POD, and fungal biomass were negatively correlated with PC2. The inoculated + IAA group aligned closely with these variables, indicating that exogenous IAA led to significant increases in IAA content, POD activity, and fungal proliferation. A correlation heatmap (Fig. 4B) showed strong positive correlations between CAT and SOD (0.83), IAA and fungal biomass (0.68), PAL and flavonoids (0.6), as well as IAA and POD, H₂O₂ and flavonoids, and POD and fungal biomass. These results indicate that IAA functionally links oxidative stress, secondary metabolic defense, and fungal proliferation, thereby altering the plant’s response to S. scitamineum. Ultimately, this led to a greater divergence in physiological patterns from the control (CK) than was observed in the inoculation-only treatment.

### Exogenous IAA modulated fungal effector gene expression

**Fig. 5:**
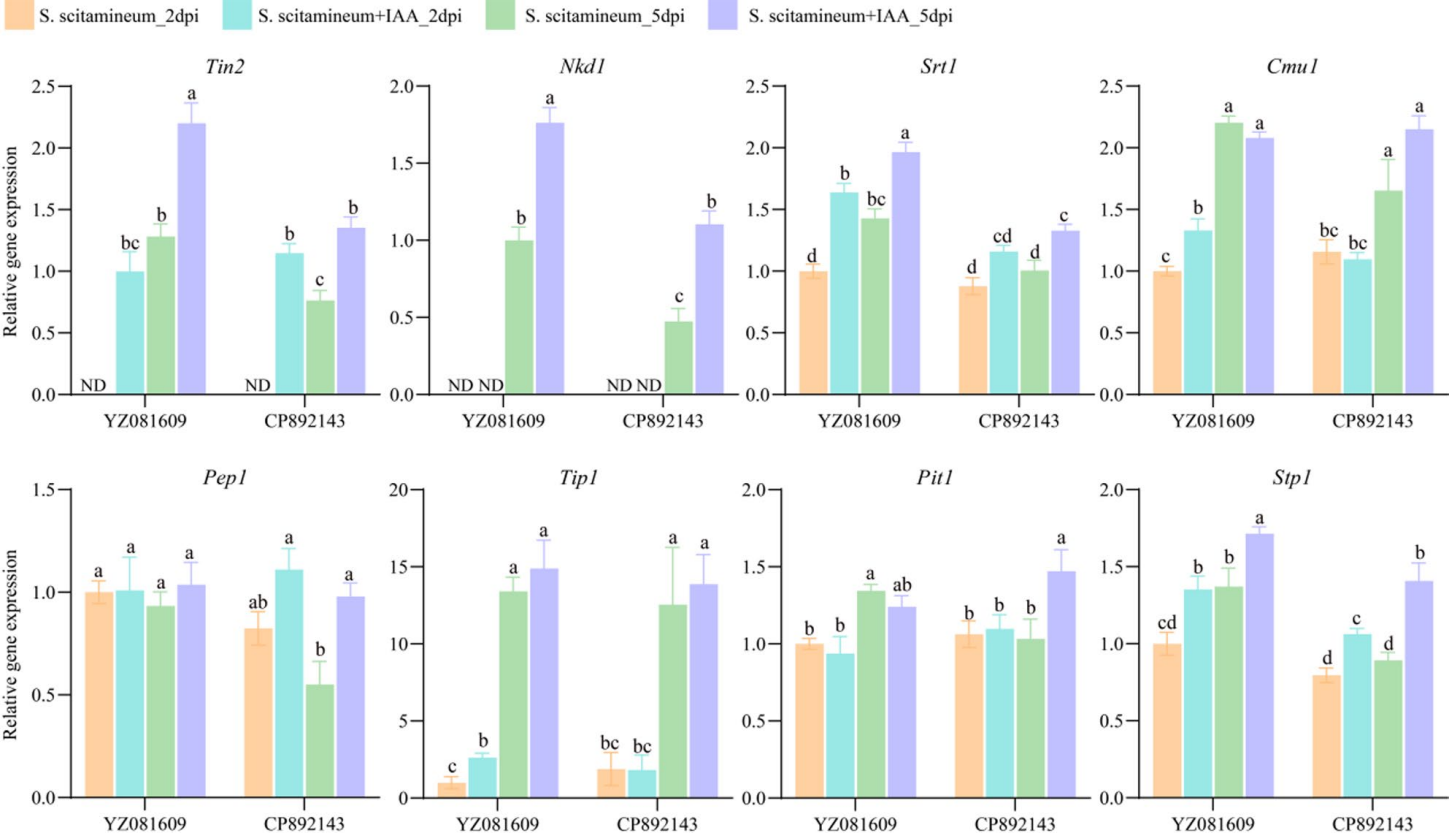
Expression patterns of eight fungal effector genes in susceptible (YZ081609) and resistant (CP892143) genotypes at 2 and 5 days post-inoculation. Expression levels were normalized to the *S. scitamineum* housekeeping gene *GAPDH*, which served as an internal reference. Data represent mean ± SD from three biological replicates with three technical replicates per biological replicate. Different lowercase letters indicate significant differences between groups (one-way ANOVA followed by LSD test; *P* < 0.05). ND, Not Detectable

We analyzed the expression of eight fungal effector genes in susceptible (YZ081609) and resistant (CP892143) genotypes at 2 and 5 dpi with *S. scitamineum*, either alone or combined with IAA treatment (Fig. 5). Most effectors (including *Tin2*, *Nkd1*, *Srt1*, *Cmu1*, *Tip1*, and *Stp1*) showed time-dependent upregulation from 2 to 5 dpi. Notably, *Tin2* and *Nkd1* were undetectable at 2 dpi under pathogen-only treatment. The resistant genotype CP892143 exhibited significantly lower expression of most effector genes (e.g., *Tin2*, *Nkd1*, *Srt1*, *Stp1*) compared to YZ081609. IAA-induced effector expression was gene-specific. In both genotypes, IAA application significantly upregulated *Tin2*, *Nkd1*, *Srt1*, and *Stp1*. For instance, *Tin2*, which was undetectable in the pathogen-only group at 2 dpi, became detectable with IAA co-application. In contrast, *Pep1* expression remained unchanged in the susceptible material but was upregulated in the resistant material at 5 dpi under pathogen + IAA. Additionally, *Cmu1* and *Tip1* expression was significantly higher in the susceptible material under pathogen + IAA at 2 dpi, whereas no significant changes occurred in the resistant material.

### Identification and characterization of key IAA-biosynthetic genes in *S. scitamineum*

To explore the genetic basis of IAA biosynthesis in *S. scitamineum*, we identified and characterized candidate genes involved in Trp-dependent auxin synthesis pathways. Genome-wide analysis revealed multiple orthologs encoding key enzymes, including two putative Trp aminotransferases (designated SsTam1 and SsTam2) and sixteen indole-3-acetaldehyde (IAAld) dehydrogenases (SsIad1–SsIad16) (Table 2). Protein sequence analysis confirmed that all identified SsTam and SsIad proteins contain characteristic functional domains: SsTam1 and SsTam2 possess the conserved aminotransferase domain (Pfam PF00155, belong to ARO8 superfamily), and all SsIad members contain the aldehyde dehydrogenase domain (Pfam PF00171, belong to ALDH superfamily) (Fig. S1). Their molecular properties were shown in Table S2.

**Table 2:** Identification of Tam and Iad orthologs in *S. scitamineum*

Protein	S. scitamineum ID	Bait	Identity (%)	E Value	Pfam
SsTam1	SPSC_03059	XP_011387757.1 Trp amino transferase [Mycosarcoma maydis]	95.03	0	PF00155.24
SsTam2	SPSC_00463		65.38	1.916E-178	PF00155.24
SsIad1	SPSC_01532	AAC49575.1 indole-3-acetaldehyde dehydrogenase [Mycosarcoma maydis]	98.99	0	PF00171.25
SsIad2	SPSC_01254		70.9	0	PF00171.25
SsIad3	SPSC_00448		68.71	0	PF00171.25
SsIad4	SPSC_01024		53.36	6.886E-89	PF00171.25
SsIad5	SPSC_00191		52.24	6.791E-104	PF00171.25
SsIad6	SPSC_01038		50.59	4.176E-71	PF00171.25
SsIad7	SPSC_00190		47.89	3.330E-103	PF00171.25
SsIad8	SPSC_05314		45.95	1.144E-56	PF00171.25
SsIad9	SPSC_00913		44.83	3.741E-62	PF00171.25
SsIad10	SPSC_03552		43.74	1.839E-42	PF00171.25
SsIad11	SPSC_06046		43.59	1.236E-53	PF00171.25
SsIad12	SPSC_02451		40.62	2.780E-34	PF00171.25
SsIad13	SPSC_00869		39.69	1.823E-29	PF00171.25
SsIad14	SPSC_05029		39.34	6.860E-41	PF00171.25
SsIad15	SPSC_05123		36.95	1.125E-46	PF00171.25
SsIad16	SPSC_00587		33.86	8.717E-27	PF00171.25

**Fig. 6:**
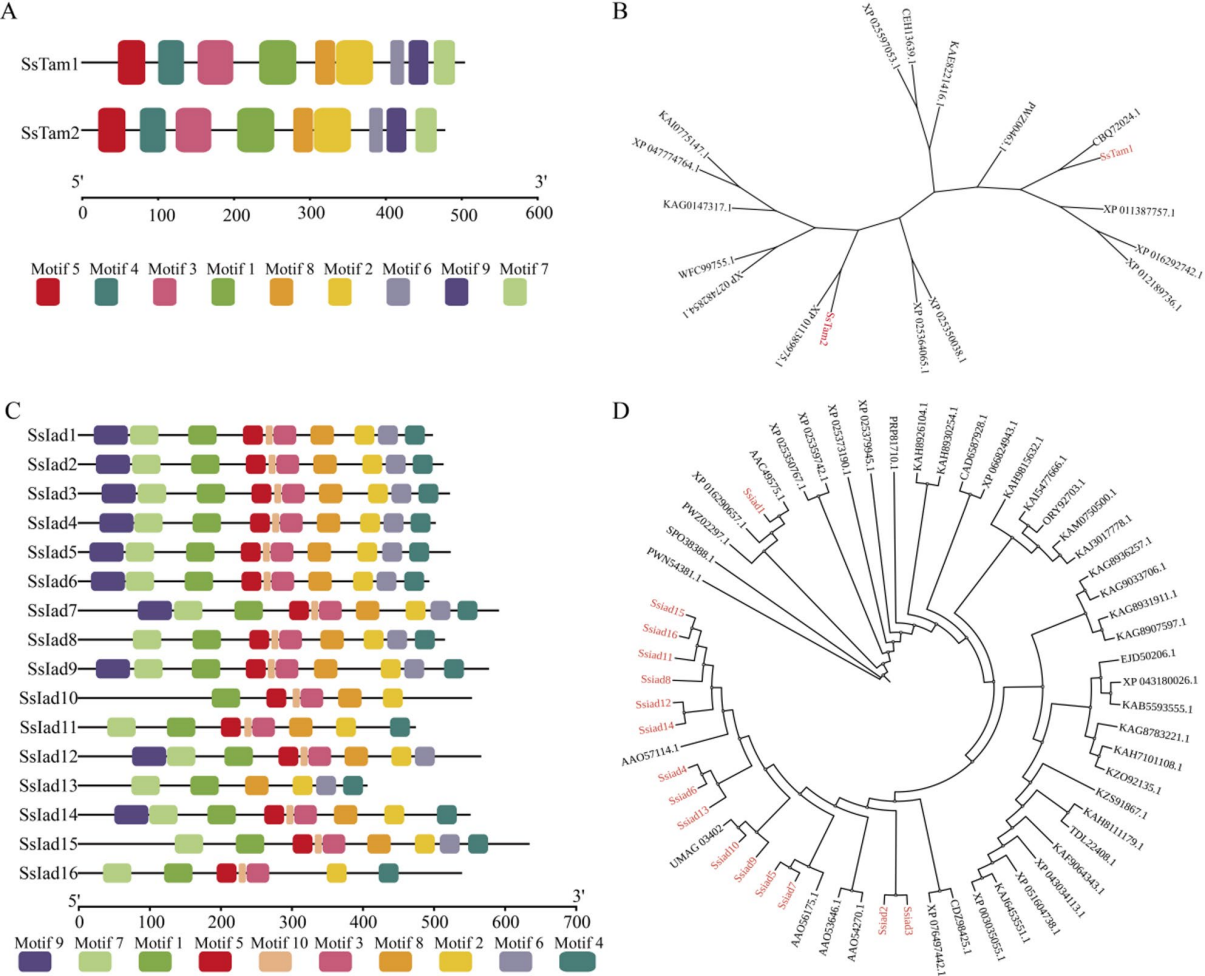
Motif composition and phylogenetic analysis of SsTam and SsIad gene families.**A** Motif composition of SsTam1 and SsTam2 proteins; different colored boxes represent distinct conserved motifs. **B** Phylogenetic tree of SsTam proteins (red labels indicate *S. scitamineum* proteins) constructed using the neighbor-joining method. **C** Motif composition of SsIad family proteins; colored boxes denote various conserved motifs. **D** Phylogenetic tree of SsIad proteins (red labels indicate *S. scitamineum* proteins) generated via the neighbor-joining method

Motif architecture analysis revealed conserved composition and sequential order of protein motifs within each family. SsTam1 and SsTam2 shared an identical motif pattern, and SsIad proteins showed high structural similarity, particularly among closely related members (Fig. 6A, Fig. S2). Phylogenetic analysis revealed that SsTam proteins form a well-supported clade with functionally characterized Trp aminotransferases from related smut fungi, including *Sporisorium reilianum* mitochondrial kynurenine/alpha-aminoadipate aminotransferase (CBQ72024.1) and *Mycosarcoma maydis* Tams (XP_011387757.1, XP_011389975.1) (Fig. 6B; Table S3). The SsIad family was divided into two distinct clades. SsIad1 clustered with functionally validated fungal Iads from *Mycosarcoma maydis* (AAC49575.1), *Kalmanozyma brasiliensis* (XP_016290657.1), and Testicularia cyperi (PWZ02297.1). The remaining SsIad2–SsIad16 formed a separate, expanded clade that exhibited close affinity not only to bacterial Iads from *Pseudomonas syringae* (AAO57114.1, AAO54270.1, AAO53646.1, AAO56175.1) [32] but also to fungal homologs including the *M. maydis* Iad (UMAG_03402). This phylogenetic distribution indicates that SsIad proteins are evolutionarily conserved across smut fungi and even share homology with bacterial lineages. The notable expansion of the SsIad family further suggests functional diversification, likely supporting adaptive roles in the interaction with sugarcane hosts. In summary, our genomic and phylogenetic analyses confirm that *S. scitamineum* possesses a complete set of candidate genes potentially involved in IAA biosynthesis via Trp-dependent pathways. These findings provide a genetic basis for the fungus’s ability to synthesize and possibly manipulate auxin levels during host interaction.

### Specific IAA-biosynthetic gene expression is associated with host colonization

**Fig. 7:**
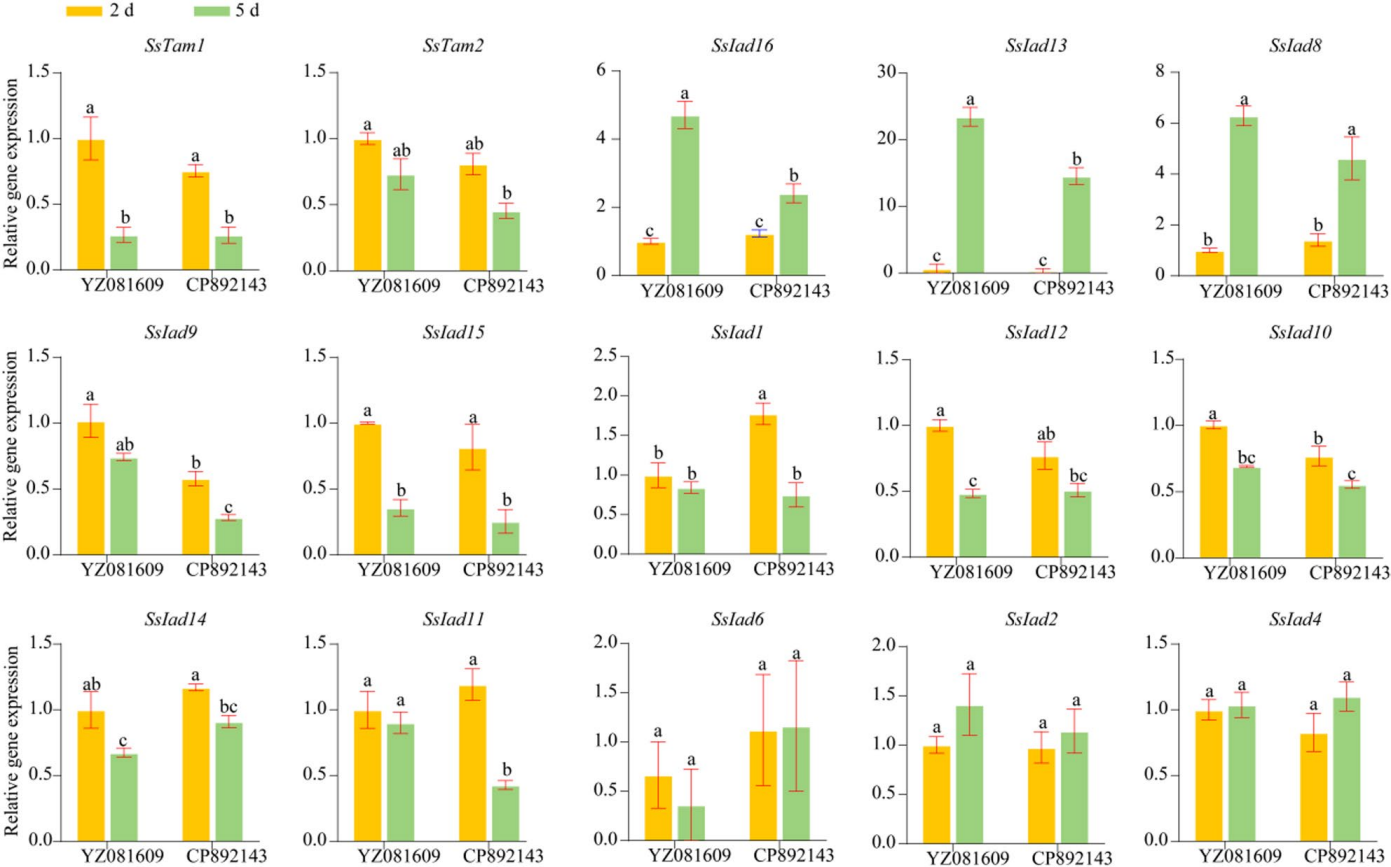
Relative expression levels of 15 IAA-biosynthetic genes in susceptible (YZ081609) and resistant (CP892143) genotypes at 2 (yellow) and 5 (green) dpi. Expression levels were normalized to the *S. scitamineum* housekeeping gene *GAPDH*, which served as an internal reference. Data represent mean ± SD from three biological replicates with three technical replicates per biological replicate. Different lowercase letters indicate significant differences between groups (one-way ANOVA followed by LSD test; *P* < 0.05)

To assess whether the identified IAA-biosynthetic genes are functionally involved in infection, we analyzed their expression profiles during colonization of susceptible (YZ081609) and resistant (CP892143) sugarcane varieties. Among the 18 genes analyzed, *SsIad3*, *SsIad5*, and *SsIad7* were undetectable under all conditions. Detectable genes exhibited distinct temporal and genotype-specific regulation (Fig. 7). A subset, including *SsTam1*, *SsIad15*, *SsIad14*, and *SsIad10*, was significantly downregulated at both time points in both varieties. In contrast, *SsIad16*, *SsIad13*, and *SsIad8* were identified as late-infection-induced genes, with expression strongly upregulated at 5 dpi in YZ081609; for example, *SsIad13* expression increased more than 30-fold. Several genes showed genotype-dependent expression. *SsIad12* was downregulated over time specifically in the susceptible host, while *SsIad1*, *SsIad9*, *SsIad10*, and *SsIad11* were downregulated in the resistant genotype CP892143. Conversely, *SsIad2*, *SsIad4*, and *SsIad6* expression remained stable across genotypes and time points. These results indicate that fungal IAA-biosynthetic genes are dynamically regulated in a time- and host-dependent manner during infection. Genes such as *SsIad13* and *SsIad16* were strongly induced late in the susceptible host, whereas the resistant host triggered downregulation of a distinct gene set, suggesting specialized roles in colonization and host defense evasion.

## Discussion

### The dual role of IAA in the sugarcane–smut interaction

Recent studies have elucidated the molecular mechanisms by which IAA functions in plant–pathogen interactions, particularly the strategies employed by plant pathogens to manipulate auxin homeostasis in order to facilitate infection [41]– [42]. Research had shown that a localized increase in IAA levels during the early stages of interaction with host plants was a key factor promoting plant susceptibility to infection by bacterial pathogens such as *Pseudomonas syringae* [43]– [44]. Consistently, we found that pretreatment with 0.5 mM IAA significantly promoted subsequent fungal colonization and disease development in planta. This exogenous application raised the shoot IAA content to approximately 110 ng·g⁻¹ FW ( about 0.6 µM, *Table **S1*), a level we selected to mimic and moderately amplify the pathological auxin response. Specifically, this induced concentration lay within the upper range of endogenous IAA levels observed during natural infection (about 0.2 µM), and was substantially higher than basal levels (about 0.05–0.1 µM), yet far below the directly inhibitory concentrations (≥ 1 mM) effective in vitro. These data support the conclusion that IAA facilitates infection primarily by modulating host physiology within a defined, pathologically relevant concentration range, rather than by directly stimulating the pathogen Such a strategy aligns with reports in other smut fungi, like *U. maydis*, which employs effectors or its own IAA biosynthesis to interfere with host auxin signaling [34, 38]. Of particular note, the direct growth inhibition by high IAA underscores the need for precise regulation of IAA metabolism by the pathogen itself. While the precise regulatory mechanisms in *S. scitamineum* remain unknown, studies in bacterial pathogens like *P. savastanoi* had shown that dedicated IAA-conjugating enzymes (e.g., IAA-lysine synthase) were crucial to prevent toxic overaccumulation [40]. Thus, it is plausible that fine-tuning of IAA levels presents a challenge for the smut pathogen, balancing its virulence function against potential costs.

### IAA-mediated perturbation of host redox homeostasis and defense metabolism

The maintenance of redox homeostasis and the activation of defense metabolism are crucial components of plant immunity against pathogen invasion [45]– [46]. Our analysis revealed that IAA was significantly positively correlated with fungal biomass and POD activity, while also linking oxidative stress (H₂O₂) with secondary metabolism (flavonoids). These relationships suggest that IAA functionally connects oxidative stress, fungal proliferation, and the plant’s metabolic defense response. The accumulation of H₂O₂, likely resulting from a pathogen-induced oxidative burst, may act as a signaling cue to activate plant defense mechanisms. This is supported by the transient induction pattern of the antioxidant enzymes SOD and CAT, which peaked early (at 2 dpi) but were not sustained—particularly in the resistant genotype CP892143. Such a pattern implies a potential suppressive role of IAA in the plant’s basal antioxidant defense over time, which aligns with findings from the *U. maydis* system, where exogenous auxin was shown to reduce antioxidant enzyme activities [47]. The resistant CP892143 exhibited strong, sustained induction of CAT activity during early infection, maintained higher PAL activity, and consistently elevated basal flavonoid levels. In contrast, the susceptible YZ081609 displayed delayed and transient defense activation with weaker antioxidant responses and flavonoid accumulation, thereby illustrating the crucial role of timely and robust physiological defense in determining disease resistance. Taken together, these findings demonstrate that IAA contributes to susceptibility by disrupting redox homeostasis and flavonoid-based defense metabolism, with the host’s genetic constitution playing a decisive role in determining the ultimate outcome of the plant-pathogen interaction.

### Fungal effector responses to IAA signaling

Fungal effectors critically mediate fungus–plant interactions by subverting host immunity and physiology to facilitate colonization; studies in *U. maydis* further demonstrated their conserved roles in manipulating auxin signaling, suppressing oxidative bursts, and compromising cell wall integrity [48–50]. Here, we present novel evidence suggesting that IAA signaling modulates effector expression in *S. scitamineum*, uncovering a novel dimension of crosstalk between phytohormones and fungal pathogenicity. Exogenous IAA substantially upregulated key effector genes—including *Tin2*, *Nkd1*, *Srt1*, and *Stp1*. Notably, *Tin2*, nearly undetectable at 2 dpi under pathogen-only conditions, became strongly induced upon IAA co-application, implying IAA triggers fungal pathogenic programming. This regulatory pattern displayed distinct gene-specific and cultivar-dependent characteristics: for instance, *Pep1* remained stable across treatments in susceptible plants yet was upregulated uniquely under combined pathogen + IAA treatment at 5 dpi in resistant CP892143, supporting the view that IAA modulates effector expression indirectly via alteration of host immunity rather than through direct transcriptional control. Functional annotation suggests these effectors operate within an integrated virulence network: the putative *Pep1* ortholog (62.7% identical to *U. maydis Pep1*), showing early and persistent induction, likely reinforced IAA-driven antioxidant suppression by inhibiting host peroxidase activity and constraining ROS burst [51]; *Cmu1*, peaking later during infection, might synergize with IAA to antagonize salicylic acid signaling through depletion of SA precursors; *Tin2*, markedly induced at mid-late infection stages, was proposed to act synergistically with IAA-induced cell division by diverting metabolic flux from lignin toward anthocyanin production, thereby attenuating structural defenses [52]; meanwhile, sustained upregulation of the sugar transporters *Srt1* and *Stp1* reflects ongoing host carbon reprogramming, further amplified by IAA to bolster nutrient acquisition. Marked host genotype-specific expression patterns were also observed—certain effectors were uniquely induced late in susceptible cultivars, potentially tied to sporulation, whereas others were elevated early in resistant plants, possibly in response to ROS induction—illustrating that effector expression is shaped not only by pathogen development but also decisively by host immune context [53]– [54]. Together, our results demonstrate that the pathogen orchestrates IAA signaling and effector expression in a coordinated manner to promote disease. Our findings suggest that IAA-mediated host susceptibility is associated with effector-driven virulence. A key unresolved question is whether IAA regulates effector expression directly via fungal signaling or indirectly through host physiological changes. Future work should employ genetic and mechanistic approaches using in vitro binding and protein-protein interaction assays to characterize potential interactions between IAA signaling components and fungal effector.

### Dynamic regulation of IAA biosynthetic genes in Smut fungus during host interaction

Microbial auxin biosynthesis via Trp-dependent pathways is well-established in numerous plant-associated microbes. In *U. maydis*, the indole-3-pyruvic acid (IPA) pathway involves Tam1/Tam2, which catalyze Trp transamination, and Iad1/Iad2, responsible for converting IAAld to IAA [55]– [56]. Similarly, in *Magnaporthe oryzae*, MoTam1 and MoIpd1 operated within the IPA pathway to support fungal growth and pathogenicity [57]. Our genomic analysis confirms that *S. scitamineum* retains a complete IPA pathway gene set, encoding two *Trp aminotransferases* (*SsTam1/SsTam2*) and sixteen *IAAld dehydrogenases* (*SsIad1–16*). Of particular note, *SsTam1* corresponds to the previously identified *SsAro8*, which is essential for fungal mating, filamentation, and virulence [36], thereby functionally linking IAA biosynthesis with developmental processes in the fungus.

Expression profiling during host infection revealed intricate temporal and genotype-dependent regulation of these biosynthetic genes. *SsTam1* was generally downregulated, especially in the resistant cultivar CP892143, implying possible host-mediated suppression of fungal primary metabolism and potential fungal reliance on host-derived IAA. This aligns with observations in *P. syringae*, where bacterial auxin synthesis aided virulence but remained under host regulatory influence [31]– [58]. Among Iad family members, *SsIad3*, *SsIad5*, and *SsIad7* were consistently undetectable, while *SsIad15*, *SsIad14*, and *SsIad10* were markedly downregulated—a pattern suggestive of metabolic conservation. Strikingly, *SsIad13* and *SsIad16* were strongly induced (over 30-fold) during late infection specifically in susceptible YZ081609, indicating a specialized role in sustaining IAA homeostasis during biotrophic establishment. Such functional specialization among Iad homologs mirrors findings in *U. maydis*, where different members contribute variably to virulence [55]. The dual metabolic role of SsTam1/SsAro8 in IAA and tryptophol biosynthesis designated it as a key regulatory node, consistent with reports in other fungi where auxin pathway enzymes often fulfilled pleiotropic metabolic roles [57, 59]. The stage- and genotype-specific expression patterns of IAA biosynthetic genes likely reflect an adaptive strategy to optimize IAA-mediated benefits while minimizing its costs, paralleling regulatory mechanisms observed in other pathosystems [33, 60]. Future studies should prioritize functional validation via genetic tools and dissect the respective contributions of plant and fungal IAA synthesis during infection, building on insights from established model systems. While our expression data suggest the fungal IPA pathway is dynamically regulated during infection, the present study cannot distinguish the relative contributions of host- versus pathogen-derived IAA to the hormonal pool manipulating host physiology. To this end, future studies should create a pathogen mutant deficient in IAA synthesis (e.g., *ΔTam*, *ΔIad*) to directly quantify the pathogen’s contribution, while simultaneously employing plant auxin-responsive reporters and transcriptomics to monitor the activation of the host’s own IAA biosynthesis and signaling pathways in response to infection by both wild-type and mutant strains. Furthermore, the proposed roles of these fungal IAA-biosynthesis genes, inferred from expression patterns and homology, require functional validation through targeted gene modification, complementation in *S. scitamineum* and biochemical assays of their activity.

## Conclusions

**Fig. 8:**
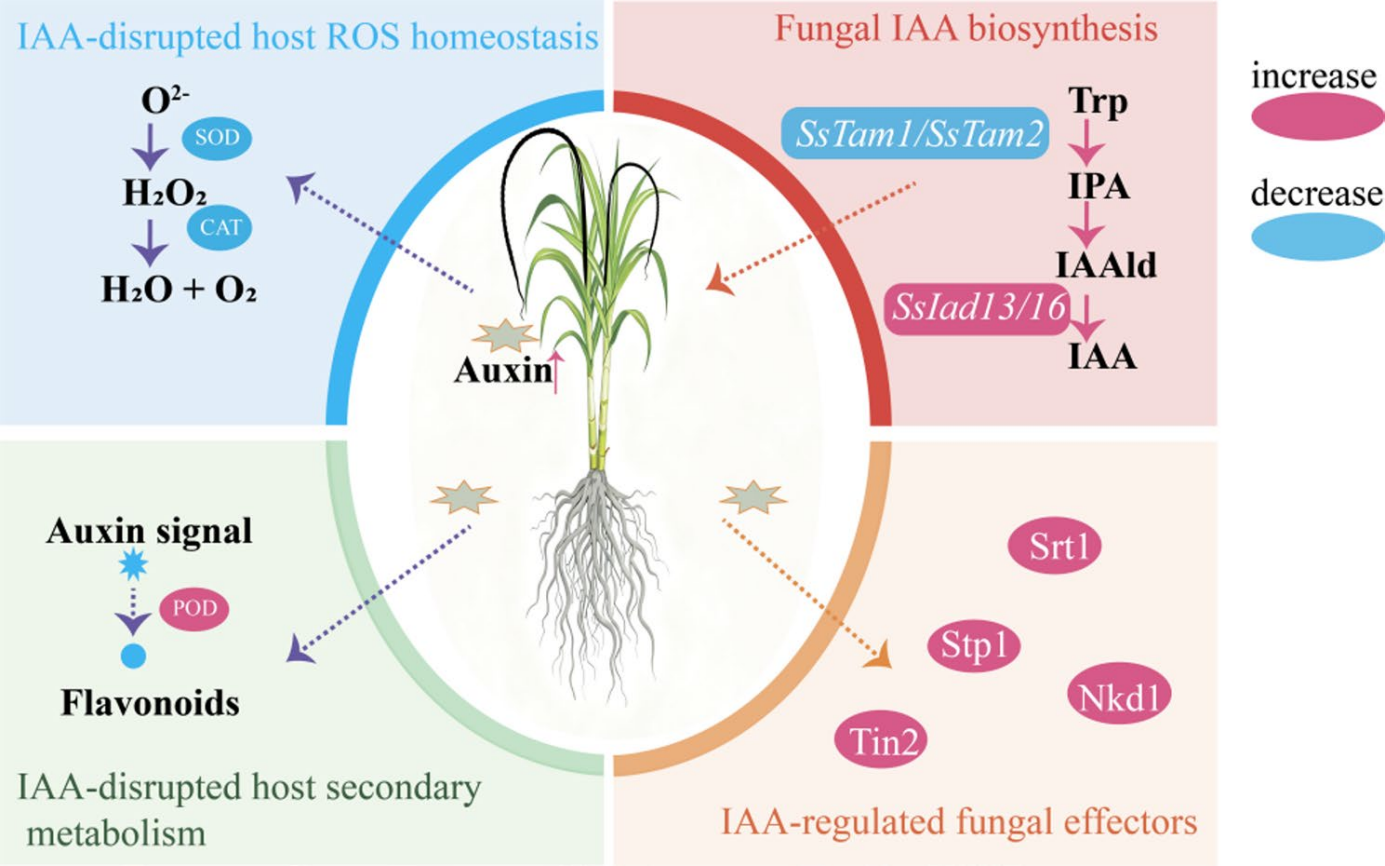
A working model of the IAA-mediated regulatory network during the early interaction between sugarcane and *S. scitamineum*. Red and blue ovals indicate increased and decreased levels of the corresponding components, respectively

This study provides correlative molecular and physiological evidence supporting a significant role for auxin in the *S. scitamineum*–sugarcane interaction, with its manipulation likely constituting an important virulence mechanism. We showed that IAA promoted infection in a dose-dependent manner, reprogramming host physiology at low concentrations in planta despite inhibiting growth at high levels in vitro. This reprogramming involved coordinated disruption of ROS homeostasis via suppression of SOD and CAT activities, attenuation of flavonoid-mediated defense, and modulation of key effector genes (e.g., *Srt1*, *Stp1*, *Tin2*, *Nkd1*) (Fig. 8). To optimize virulence while minimizing self-toxicity, the pathogen dynamically regulated IAA biosynthetic genes (*SsTam1*, *SsIad13/16*) in temporal and host genotype-specific patterns. Ultimately, the host’s genetic background acted in concert with IAA activity to shape infection outcomes: while IAA elevated colonization in both cultivars, the resistant genotype’s constitutive defenses effectively restricted disease progression. Together, these results support a model in which auxin signaling aligns effector deployment with host physiological reprogramming, and fungal IAA biosynthetic components identified here represent candidate targets whose validation could inform future strategies for smut control.

## Materials and methods

### Spore germination assay and culture

*S. scitamineum* used in this study was originally isolated from infected whip-like sori of the susceptible sugarcane cultivar ROC22 in Kaiyuan City, Yunnan Province, China, in 2023. Teliospores were collected, air-dried at room temperature (25 ± 2 °C) for 7 days, and stored in sealed centrifuge tubes at 4 °C until further use. Prior to experimental use, spore viability was assessed on 1% water agar plates. Teliospores were evenly spread on the medium and incubated at 28 °C in darkness for 24 h; only batches exhibiting a germination rate exceeding 90% (based on counts of at least 300 spores per plate under a light microscope) were selected for subsequent assays. To evaluate the effect of IAA on spore germination, teliospores were plated on 1% water agar supplemented with four different IAA (Sigma-Aldrich, St. Louis, MO, USA) concentrations (0, 0.5, 1, and 2 mM), followed by incubation at 28 °C in the dark for 8 h. Germination rates were determined microscopically using the same counting threshold. Each treatment consisted of three biological replicates. For time-resolved analysis of mycelial growth, teliospores were first germinated and grown on solid YEBP medium for 5 days. The resulting mycelia were then transferred into 50 mL of liquid YEBP medium containing eight IAA concentrations (0, 0.005, 0.01, 0.05, 0.1, 0.5, 1, and 2 mM) and cultured for six time intervals (6, 12, 18, 21, 24, and 30 h). Each treatment was replicated three times, and optical density at 600 nm (OD₆₀₀) was measured twice per replicate.

### Plant materials and treatments

Two sugarcane cultivars (*Saccharum* spp. hybrid) with contrasting smut resistance responses were employed in this study: the resistant genotype CP892143 and the susceptible genotype YZ081609, both provided by the Sugarcane Research Institute, Yunnan Academy of Agricultural Sciences. Field inoculation trials over three consecutive years confirmed distinct disease phenotypes, with CP892143 exhibiting an average smut incidence of approximately 3%, compared to about 48% in YZ081609. Healthy stalks of each cultivar were selected and cut into single-bud setts, which were surface-cleaned by immersion in sterile flowing water for 24 h. The setts were then transferred to a growth chamber set at 32 °C, 80% relative humidity, and a 16 h/8 h light/dark cycle until buds sprouted and reached approximately 2 cm in length [7]. Before inoculation, germinated buds were immersed in 0.5 mM IAA solution for 30 min, while control buds were treated with sterile water. After 24 h of IAA pretreatment, an inoculum was prepared by suspending qualified teliospores in sterile distilled water containing 0.01% (v/v) Tween-20 to a final concentration of 5 × 10⁶ spores/mL. Buds were inoculated with 10 µL of spore suspension delivered into the bud using a sterile 200 µL micropipette tip (0.5 mm diameter). Control buds received 10 µL of sterile 0.01% Tween-20 solution via the same puncture method. Each treatment—including uninoculated control, pathogen-only, and pathogen + IAA groups—comprised at least forty setts. Following inoculation, all plants were maintained in a growth chamber at 28 °C, 80% relative humidity, under a 16 h/8 h photoperiod. For histological examination of *S. scitamineum* colonization in sugarcane, bud samples collected at 5 days post-inoculation were transversely and longitudinally sectioned using a surgical blade. The sections were subsequently stained with 0.05% toluidine blue (pH 4.3) and digitally imaged under an Olympus BX43 microscope (Leica Microsystems) [2]. For subsequent molecular and physiological assays, inoculated buds were collected at 2 and 5 dpi. Each sample consisted of five pooled buds from independent biological replicates, immediately frozen in liquid nitrogen, and stored at − 80 °C until analysis. The entire experiment was conducted with three independent biological replicates for all downstream assessments.

### Quantification of *Sporisorium Scitamineum* biomass by qPCR

Genomic DNA was extracted from 100 mg of sugarcane bud tissue using the FastPure^®^ Plant DNA Isolation Mini Kit (Vazyme Biotech Co., Ltd., Nanjing, China) according to the manufacturer’s instructions, with minor modifications. Fungal biomass in planta was quantified by qPCR as described previously [61], using a recombinant plasmid pMD18-T-bE carrying the bE gene as the quantitative standard. A standard curve was generated from 10-fold serial dilutions of the plasmid, yielding the regression equation y = − 3.188x + 26.540, with an amplification efficiency of 105.928% (R² = 0.999). Reactions were performed in a 20 µL system consisting of 10 µL of 2× ChamQ Universal SYBR qPCR Master Mix (Vazyme Biotech Co., Ltd., Nanjing, China), 1.0 µL each of forward and reverse primers (10 µM, Table S4) targeting the bE gene, 2.0 µL of DNA template, and nuclease-free water to volume. Each qPCR run included the plasmid as a positive control, and all test samples contained 2 µL of genomic DNA extracted from the respective treatment groups.The thermal cycling protocol comprised an initial denaturation at 95 °C for 2 min, followed by 40 cycles of 95 °C for 10 s and 60 °C for 30 s. A melting curve analysis was subsequently performed by heating from 60 °C to 90 °C at a rate of 0.15 °C/s, with continuous fluorescence acquisition. Fungal DNA copy numbers were calculated based on the formula established by Su [62]. The experiment consisted of three independent biological replicates, each comprising three technical replicates.

### Quantification of endogenous IAA, flavonoids, and antioxidant enzyme activities

Endogenous IAA was extracted and quantified according to a previously established method [63]. Briefly, approximately 0.4 g of frozen tissue was homogenized in 1.5 mL of pre-cooled 80% methanol and incubated for 24 h at 4 °C. After centrifugation (8000 × *g*, 10 min, 4 °C), the supernatant was collected, and the residue was re-extracted with 0.5 mL of the same solvent for 2 h. Combined supernatants were concentrated under a nitrogen stream, acidified to pH 2 with 1 M citric acid, and partitioned twice against ethyl acetate. The organic phase was dried, reconstituted in 0.2 mL methanol, and analyzed by HPLC (Waters 2695 system) using a Compass C18(2) column (250 × 4.6 mm, 5 μm) with an isocratic mobile phase of water/1% formic acid (65:35, v/v) at 1 mL/min and 30 °C. IAA was detected at 275 nm. The experiment consisted of three biological replicates.

Total flavonoid content was determined using a plant flavonoid assay kit (Suzhou Michy Biomedical Technology Co., Ltd) following established protocols [64]. Dried and powdered samples (0.05 g) were extracted in 1 mL of 60% ethanol at 60 °C for 2 h. After centrifugation (10,000 × *g*, 10 min), 80 µL of supernatant was mixed with an equal volume of 60% ethanol, incubated for 15 min at room temperature, and absorbance was measured at 510 nm. For H_2_O_2_ measurement and enzyme assays, approximately 0.3 g of fresh sheath tissue was ground in liquid nitrogen and homogenized in extraction buffer (1 mL per 0.1 g tissue). The homogenate was centrifuged (12,000 × *g*, 10 min, 4 °C), and the supernatant was used for subsequent analyses. H₂O₂ content and the activities of PAL, POD, SOD, and CAT were determined using commercial assay kits (M0107A, M0110A, M0105A, M0101A, M0103A; Suzhou Michy Biomedical Technology). H₂O₂ concentration was measured at 415 nm according to the titanium-peroxide method [65]. PAL [66], POD [67], SOD, and CAT [68] activities were assessed following published procedures, respectively. The experiment consisted of three biological replicates.

### Gene expression analysis by qRT-PCR

Total RNA was extracted from sugarcane samples using the FastPure Plant Total RNA Isolation Kit (Vazyme Biotech Co., Ltd., Nanjing, China) and reverse-transcribed into cDNA with HiScript III RT SuperMix for qPCR (Vazyme Biotech Co., Ltd.). Gene-specific primers were designed and synthesized by Beijing Genome Institute (Shenzhen, China); their sequences are provided in Supplementary Table S4. qPCR was performed in 20 µL reactions containing 10 µL FastStart Universal SYBR Green Master Mix, 0.4 µL of each primer, and 2 µL cDNA template. The thermal cycling protocol consisted of initial denaturation at 95 °C for 2 min, followed by 40 cycles of 95 °C for 10 s and 60 °C for 30 s. All reactions were run in triplicate on three biologically independent samples per condition. The *S. scitamineum* house keeping gene *GAPDH* was used as an internal reference for normalization, and relative gene expression levels were calculated using the 2^−ΔΔCT^ method [69]. The experiment consisted of three independent biological replicates, each comprising three technical replicates.

### Identification and characterization of key IAA-biosynthetic genes

The genome of *S. scitamineum* strain SscI8 (Taxonomy ID: 49012; GenBank Assembly ASM90000236v1) was retrieved from the National Center for Biotechnology Information (NCBI). To identify putative TAM and IAD homologs in *S. scitamineum*, the amino acid sequences of *Mycosarcoma maydis* Tam1 (XP_011387757.1) and Iad1 (AAC49575.1) were used as queries for BLASTP searches against the fungal proteome with default parameters. Candidate sequences were filtered using an E‑value cutoff of 1 × 10⁻¹⁰, and only the longest transcript per locus was retained in cases of multiple splicing variants. All candidate proteins were further validated using the NCBI Conserved Domain Database (CDD-62456 PSSMs) and the PFAM database 38.0 (http://pfam.xfam.org/) to confirm the presence of characteristic functional domains. Physicochemical properties of the identified SsTam and SsIad proteins—including molecular weight, theoretical isoelectric point (pI), amino acid composition, instability index, and grand average of hydropathicity (GRAVY)—were predicted using the ExPASy ProtParam tool (https://www.expasy.org/protparam/). Conserved protein motifs were identified with the MEME suite (Version 5.5.9; http://meme-suite.org/tools/meme) [70] under the following settings: maximum number of motifs = 6, motif width range = 10–50 residues, and at most one occurrence per sequence. The resulting motifs and gene structures were visualized using TBtools (Version 2.097) [71]. Multiple sequence alignments of 18 Tam and 60 Iad protein sequences (listed in Table S3) were generated separately using MUSCLE in MEGA 11 under default parameters [72]. Phylogenetic trees were subsequently constructed from the alignments using the neighbor‑joining method in MEGA 11, with the Poisson correction model, pairwise deletion, and 1000 bootstrap replicates.

### Data statistics

Data processing, PCA, and subordinate function calculation were conducted using Excel (Office 2013) and IBM SPSS Statistics (version 17.0).

## Supplementary Information

Supplementary Material 1.

Supplementary Material 2.

## Data Availability

All data generated or analyzed during this study are included in this published article.

## Abbreviations

ALDH: Aldehyde dehydrogenase
ANOVA: Analysis of variance
CAT: Catalase
ET: Ethylene
IAA: Indole-3-acetic acid
IAD: IAAld dehydrogenase
JA: Jasmonic acid
LSD: Least significant difference
MDA: Malondialdehyde
PAL: Phenylalanine ammonia-lyase
PCA: Principal component analysis
POD: Peroxidase
qPCR: Quantitative real-time PCR
RDV: Rice dwarf virus
SA: Salicylic acid
SD: Standard deviation
SOD: Superoxide dismutase
TAL: Tyrosine ammonia-lyase
TAM: Tryptophan (Trp) aminotransferase
Tips: TOPLESS (TPL)-interacting protein effectors
TMV: Tobacco mosaic virus
Trp: Tryptophan
