# Supplementary material for: Auxin dysregulation: a key early event in sugarcane susceptibility to Sporisorium scitamineum
Source: BMC Plant Biol. 2026 Jan 24;26:333. doi: 10.1186/s12870-026-08187-5 (PMC12914901; doi:10.1186/s12870-026-08187-5)
Supplement: Supplementary file 1 — Supplementary Material 1. [file 12870_2026_8187_MOESM1_ESM.docx]

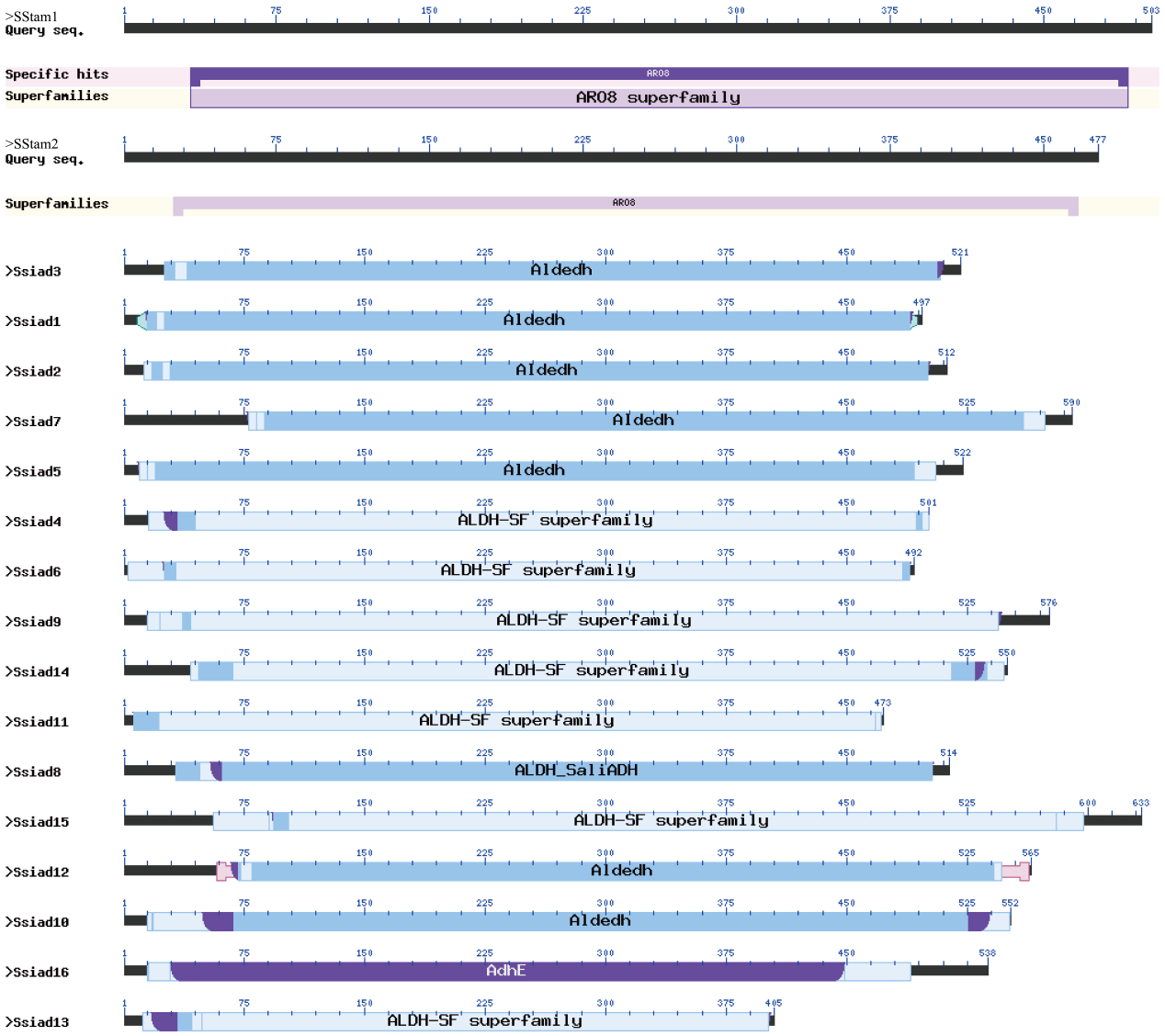


**Fig.S1** Domain architecture analysis of *SsTam1/2* and *Ssiad* gene family members by NCBI CDD tool.


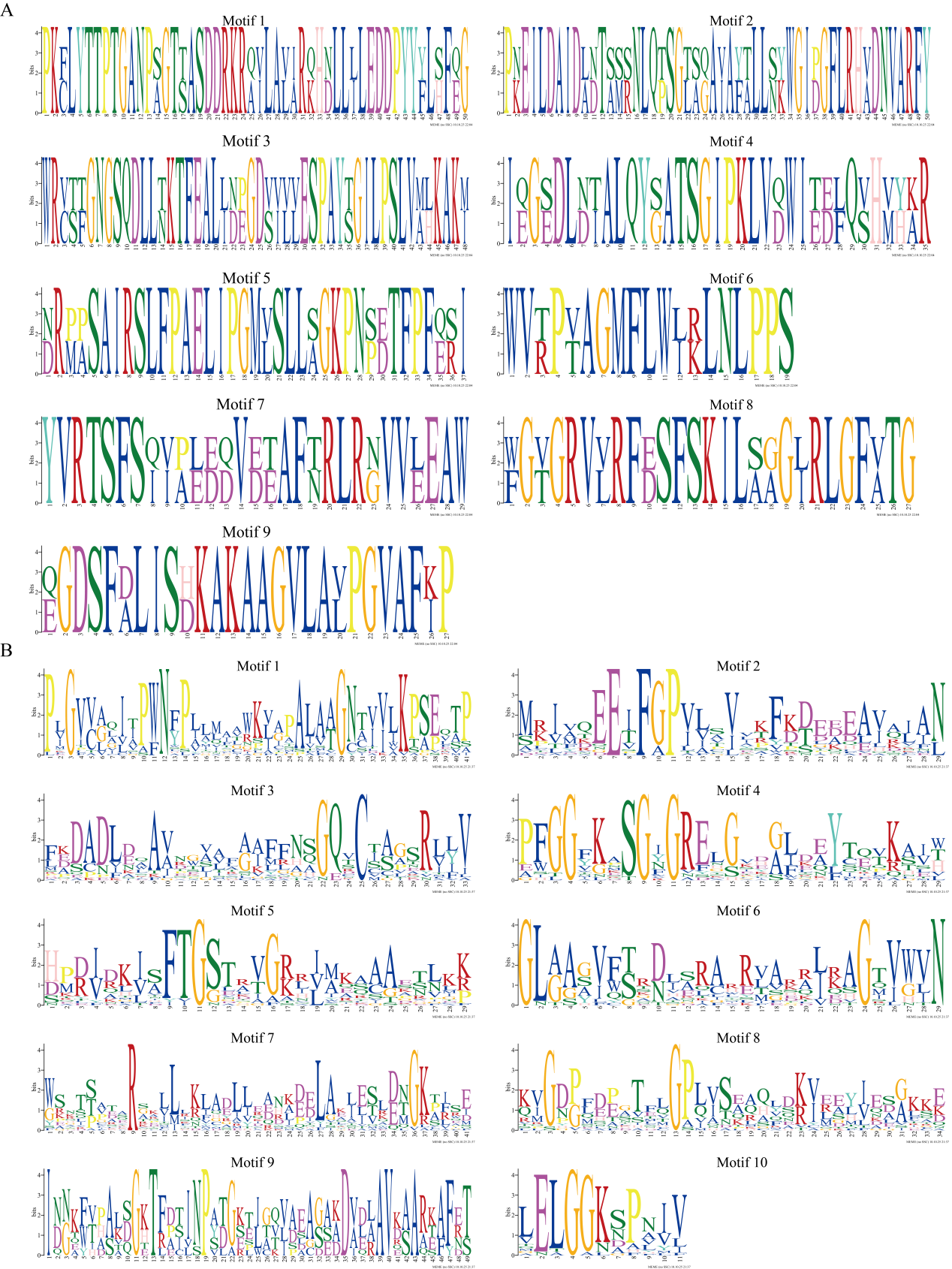


**Fig.S2** Conserved domains of SsTam and SsIad family proteins identified by the online tool MEME.
